# Comparison of Pathologic Complete Response Rates and Oncologic Outcomes in Patients With Surgically Resectable Esophageal Cancer Treated With Neoadjuvant Chemoradiation to 50.4 Gy vs 41.4 Gy

**DOI:** 10.7759/cureus.19233

**Published:** 2021-11-03

**Authors:** Anthony D Nehlsen, Eric J Lehrer, Lucas Resende-Salgado, Kenneth E Rosenzweig, Michael Buckstein

**Affiliations:** 1 Radiation Oncology, Icahn School of Medicine at Mount Sinai, New York City, USA

**Keywords:** pathologic complete response (pcr), chemoradiotherapy (chemo-rt), carcinoma, esophageal carcinoma, neo-adjuvant

## Abstract

Background

Excellent outcomes and high rates of pathologic complete response (pCR) have been reported in patients with operable esophageal carcinoma using 41.4 Gy of radiation with concurrent carboplatin and paclitaxel. With pCR rates similar to studies using higher doses, it remains unclear whether doses greater than 41.4 Gy result in improved outcomes. This study aims to compare pCR rates and oncologic outcomes in patients treated with neoadjuvant chemoradiation to 50.4 Gy vs 41.4 Gy.

Methods

We reviewed the charts of patients with operable esophageal carcinoma who were treated with neoadjuvant chemoradiation followed by oncologic resection. Our primary endpoint was the pCR rate. Secondary endpoints were overall survival, progression-free survival (PFS), and toxicity.

Results

We identified 43 patients meeting inclusion criteria. Nineteen patients were treated with 41.4 Gy and 24 were treated with 50.4 Gy. Cohorts were well-matched, except for a significantly higher percentage of patients with adenocarcinoma (AC) (89.5% vs 54.2%, p = 0.02), usage of intensity-modulated radiation therapy (IMRT) (100% vs 47.6%; p = 0.002), and usage of carboplatin, plus paclitaxel (100% vs 75%; p = 0.003) in the 41.4 Gy group. The pCR rate for the cohort was 44.2%. No differences in the pCR rate (41.7% vs 47.4%), three-year overall survival (OS) (73.7% vs 77.5%), or three-year PFS (52.8% vs 43.7%) were observed. Late toxicity rates also did not vary significantly (p = 0.2). No grade 4 or 5 events were observed.

Conclusion

In this small series, there were no differences in the pCR rate, PFS, or OS between those treated with 50.4 Gy and 41.4 Gy. Larger, multi-institutional series are needed to validate these findings.

## Introduction

Esophageal cancer remains a significant cause of cancer mortality in the United States, with the National Center for Biotechnology Information estimating 17,650 new cases and 16,080 deaths related to the disease in the year 2019 [1]. Neoadjuvant chemoradiotherapy (CRT), followed by surgical resection, is widely considered the standard of care in the management of locally advanced esophageal cancer and has been evaluated in numerous clinical trials, including the ChemoRadiotherapy for Oesophageal cancer followed by Surgery Study (CROSS), which showed excellent pathologic complete response (pCR) rates, improved overall survival (OS) and progression-free survival (PFS) in patients undergoing neoadjuvant CRT followed by surgery versus surgery alone [2].

Pathologic complete response (pCR) rates are strongly correlated with improved OS and negatively correlated with the development of local recurrence and distant metastases [3-5]. Therefore, in addition to being an objective marker of treatment response, pCR rates also appear to be a good indicator of patient outcomes. Prior to the CROSS trial being published in 2015, most patients with locally advanced esophageal cancer in the United States were treated neoadjuvant to 50 - 50.4 Gray (Gy) with concurrent chemotherapy. pCR rates of 20% - 30% were typically achieved using this technique [6-9]. The CROSS trial, which instead used a de-escalated dose of 41.4 Gy in combination with carboplatin and paclitaxel, showed a pCR rate of 29%, similar to the historical data above [10]. A pCR rate of 49% was seen for squamous cell carcinoma (SCC).

Given the success of the CROSS trial regimen, it raises the question as to whether there is a benefit to treating patients to doses higher than 41.4 Gy, such as 50 - 50.4 Gy, with the thought that lower doses of radiation should result in decreased acute and late toxicities, reduce the time before surgery, and potentially reduce the risk for postoperative complications [2]. On the other hand, it could be argued that should the patient not ultimately undergo definitive surgical resection, doses less than 50.4 Gy may not be sufficient in the definitive setting. It is also possible that despite the results of the ill-fated Intergroup Trial 0123, which showed no benefit to dose escalation in the definitive setting, outcomes, such as pCR, may be improved with higher doses of radiation [11-12].

Currently, there has not been a randomized trial examining the optimal dose of radiotherapy delivered as part of neoadjuvant CRT. Therefore, much of the information being used to make determinations on fractionation schemes is from the retrospective analysis. In this study, we aimed to use the institutional data at a large metropolitan medical center to determine whether there was a clinical benefit to treating patients with 50.4 Gy (28 fractions of 1.8 Gy) versus 41.4 Gy (13 fractions of 1.8 Gy) with concurrent chemotherapy in the neoadjuvant setting for resected esophageal cancer.

## Materials and methods

We identified the records of patients with locally advanced, resectable esophageal adenocarcinoma (AC) or SCC treated between 2007 and 2019 in the Mount Sinai Health System and performed a retrospective analysis of the cohort. Eligible patients were at least 18 years of age, had pathologic confirmation of esophageal carcinoma, had localized disease at presentation, and were treated with neoadjuvant chemoradiotherapy prior to oncologic resection. Patients were staged with computed tomography and/or positron emission tomography based on insurance approval and/or the time period in which they were treated. All patients underwent resection. Charts were reviewed for radiation, chemotherapy, staging, pathologic, and demographic data. Staging was determined using the American Joint Committee on Cancer (AJCC) 8th edition [13].

The primary endpoint was the pCR rate in each group. Secondary endpoints were OS, PFS, and rates of late toxicity. 

Statistical analyses were conducted using R Studio, version 1.1.383 [14]. The Mann-Whitney U Test and the Fisher Exact Test were used to compare continuous and categorical variables, respectively. The Drawing Survival Curves using ggplot2, version 0.4.1 was used to conduct the Kaplan-Meier analysis [15]. Kaplan-Meier curves were generated for OS and PFS for the 41.4 Gy and 50.4 Gy arms. OS and PFS were compared using the log-rank test, where a p-value < 0.05 was considered statistically significant. The pCR was defined as a binary dependent categorical variable. Univariate logistic regression was used to assess the role of covariates as predictors of pCR and were expressed as odds ratios (OR) with its associated 95% confidence interval (CI), which were considered statistically significant for p < 0.05.

The authors are accountable for all aspects of the work in ensuring that questions related to the accuracy or integrity of any part of the work are appropriately investigated and resolved. The study was conducted in accordance with the Declaration of Helsinki (as revised in 2013). The study was approved by the Institutional/Regional/National Ethics/Committee/Ethics Board of Mount Sinai Hospital (approval GCO #14-0484).

## Results

We identified a total of 43 patients treated with neoadjuvant CRT followed by oncologic resection, 19 of which were treated with 41.4 Gy and 24 were treated with 50.4 Gy. The radiation dose selected was at the discretion of the treating radiation oncologist. Eighteen of 26 patients treated prior to 2015 were treated with 50.4 Gy, while only 6/17 were treated with 50.4 Gy after 2015. The median follow-up was 28 months. Patient demographic information is listed in Table 1. 

**Table 1:** Patient Demographics

	50.4 Gray Cohort (n = 24)	41.4 Gray Cohort (n = 19)	P-value
Age			0.67
Median	69.5 years	63.5 years	
Range	34 - 80 years	26 - 77 years	
Gender			0.74
Male	75%	68.4%	
Female	25%	32.6%	
Race			0.1
White	54.5%	78.9%	
Non-White	45.5%	21.1%	
Eastern Cooperative Oncology Group ​​(ECOG) Performance Status​			0.02
0 - 1	72.2%	100%	
2	28.8%	0%	
Smoking History			0.4
Yes	89.4%	73.6%	
No	10.6%	26.4%	
Radiation Technique			0.002
Intensity Modified Radiotherapy (IMRT)	47.6%	100%	
3-Dimensional (3D)/Other	52.4%	0%	
Chemotherapy Administered			0.003
Carboplatin + Paclitaxel	75%	100%	
Other Chemotherapy	25%	0%	
Cisplatin + 5-Fluorouracil	(15%)		
5-Fluorouracil Monotherapy	(5%)		
Docetaxel, Cisplatin, 5-Fluorouracil	(5%)		
Tumor Location			
Upper Third	16.7%	0%	0.12
Middle Third	16.7%	94.7%	0.36
Lower Third	66.7%	5.3%	0.06
Histology			
Adenocarcinoma	54.2%	89.5%	0.02
Squamous Cell Carcinoma	45.8%	10.5%	
Clinical Stage			0.54
II	45.5%	42.1%	
III	54.5%	57.9%	
Tumor Stage			
1	0%	5.3%	
2	27.3%	15.8%	.47
3	68.2%	78.9%	.32
4	4.5%	0%	.99
Tumor Grade			
1	4.5%	0%	
2	59.1%	47.4%	0.54
3	36.4%	52.6%	0.22

The cohorts were well-matched for age, gender, race, and smoking history (p > .05). There was a significant increase in the Eastern Cooperative Oncology Group (ECOG) 1 - 2 (vs ECOG 0) in the 50.4 Gy group compared to the 41.4 Gy group (28.8% vs 0%; p = 0.02). The radiation technique and concurrent chemotherapy used also differed amongst groups, with higher rates of intensity modified radiotherapy (IMRT) (100% vs 47.6%; p = 0.002) and carboplatin, plus paclitaxel (100% vs 75%; p = 0.003) in the 41.4 Gy group. There were no differences between tumor location, clinical stage, primary tumor stage, or pathologic grade between the two groups (p > 0.05). However, there was an increased frequency of AC in the 41.4 Gy cohort (88.9% vs 54.2%; p = 0.02).

The pCR rate for all patients was 44.2%. As seen in Table 2, there was no difference in the pCR rate between the 50.4 Gy group and the 41.4 Gy group (41.7% vs 47.4%; p = 0.76).

**Table 2:** Pathologic Outcomes by Histology and Dose Received

Histology	Dose	Pathologic Complete Response (pCR) Rate	P-value
All Patients	All Patients (n = 43)	44.2%	0.76
	50.4 Gy (n = 24)	41.7%	
	41.4 Gy (n = 19)	47.4%	
Squamous Cell Carcinoma (SCC)	All SCC (n = 13)	61.5%	1
	50.4 Gy (n = 11)	63.6%	
	41.4 Gy (n = 2)	50%	
Adenocarcinoma (AC)	All AC (n = 28)	35.7%	0.25
	50.4 Gy (n = 13)	23.1%	
	41.4 Gy (n = 15)	47.1%	

**Figure 1:**
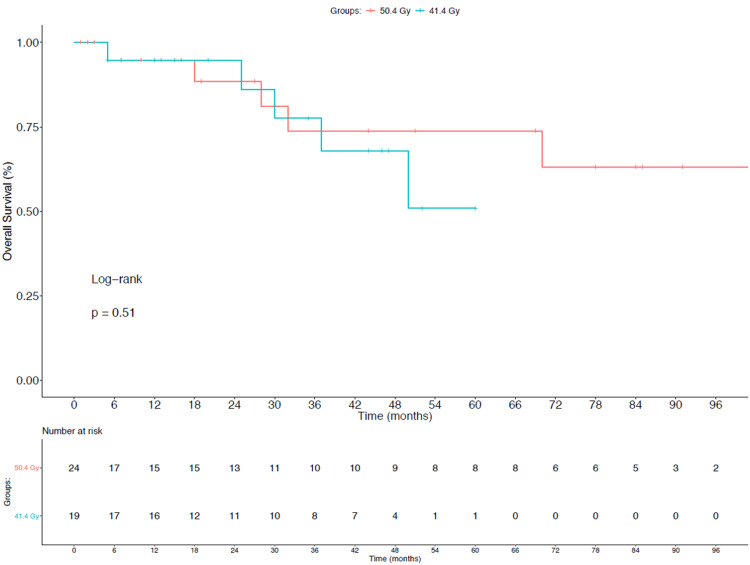
Overall Survival

For both SCC (63.6% vs 50%; p = 1.0) and AC (23.1% vs 47.1%; p = 0.25), pCR rates were not statistically different between the 50.4 Gy and 41.4 Gy groups, respectively. Additionally, there was no statistically significant difference in pCR rates in patients with SCC compared to those with AC (61.5% vs 36.7%; p = 0.18). There were no 30 or 90-day mortality events observed in either group. As seen in Figure 1, there was no difference in OS between the two groups with a three-year OS of 73.7% in the 50.4 Gy group compared to 77.5% in the 41.4 Gy group.

**Figure 2:**
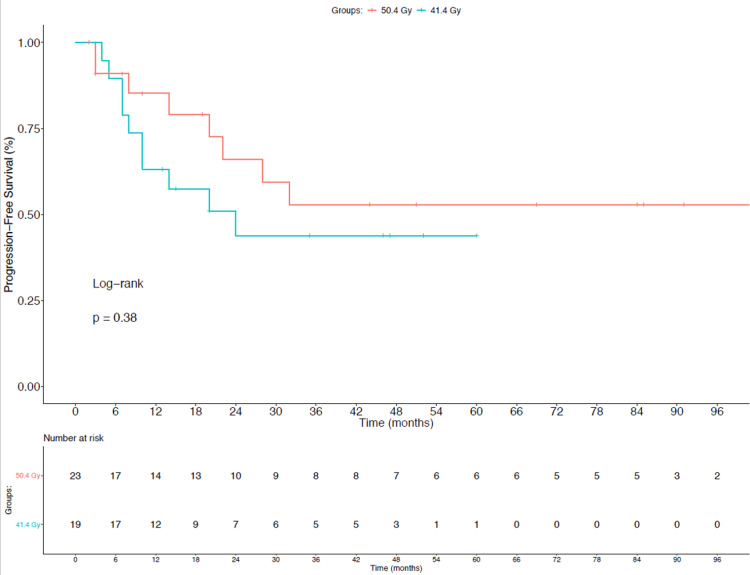
Progression-Free Survival

As seen in Figure 2, PFS was also not significantly different between cohorts at three years, with rates of 52.8% vs 43.7% in the 50.4 Gy and 41.4 Gy groups, respectively. 

Late toxicity rates did not vary significantly between the two groups, with five patients (20.8%) in the 50.4 Gy arm experiencing a grade 3 event compared to one patient (5.3%) in the 41.4 Gy group (p = 0.2). In the 50.4 Gy arm, there were three instances of stricture requiring dilation, one tracheoesophageal fistula requiring surgical correction, and one case of ARDS. The lone event in the 41.4 Gy arm was a stricture requiring dilation. No grade 4 or 5 events were observed.

In the 50.4 Gy group, 41.7% (n = 10) of patients experienced an immediate postoperative complication compared to 21.5% (n = 4) in the 41.4 Gy group (p = 0.2). The most common complications were infection (n = 7) and respiratory failure/ARDS (n = 3). The median time to surgery from the beginning of RT was 86 days (range: 66 to 305 days) in the 50.4 Gy group compared to 89 days (range: 61 to 252 days) in the 41.4 Gy group (p = 0.79). 

Univariate analysis did not identify any predictors of pCR when conducted for radiotherapy (RT) dose, histology, grade, tumor location, stage (T, N, and overall), RT technique (three-dimensional (3D) vs IMRT), smoking status, age, sex, ECOG performance status, or ethnicity. Results can be observed in Table 3.

**Table 3:** Univariate Analysis for Predictors of pCR AC: adenocarcinoma; cN Stage: clinical stage of nodes; cT Stage: clinical stage of tumor; ECOG: Eastern Cooperative Oncology Group; GEJ: gastroesophageal junction; IMRT: intensity-modulated radiation therapy; pCR: pathologic complete response; RT: radiation therapy; SCC: squamous cell carcinoma; 3D-CRT: three-dimensional chemoradiotherapy

	Hazard Ratio	95% Confidence Interval	P-value
Sex			
Male	1	1	
Female	0.45	0.11 - 1.73	0.25
Ethnicity			
Caucasian	1	1	
Non-Caucasian	0.28	0.07 - 1.04	0.06
ECOG			
0 or 1	1	1	
2+	0.9	0.13 - 7.58	0.92
Smoker			
No	1	1	
Yes	3.5	0.72 - 20	0.12
cT Stage			
T1 or T2	1	1	
T3 or T4	1.58	0.37 - 6.87	0.53
cN Stage			
N0	1	1	
N1	2.63	0.59 - 13.01	0.21
N2	6.12	0.94 - 57.28	0.07
Stage			
II	1	1	
III	3.06	0.86 - 11.75	0.09
Histology			
SCC	1	1	
AC	3.04	0.80 - 12.54	0.11
Grade			
2	1	1	
3	0.76	0.21 - 2.70	0.68
Location			
Middle or Upper	1	1	
Lower/GEJ	3.23	0.72 - 14.49	0.14
Induction Chemotherapy			
No	1	1	
Yes	0.48	0.06 - 3.21	0.45
RT Dose			
50.4 Gy	1	1	
41.4 Gy	0.79	0.23 - 2.68	0.71
RT Technique			
3D-CRT	1	1	
IMRT	1.18	0.28 - 4.83	0.82

## Discussion

The CROSS trial fully established the role of neoadjuvant CRT in the treatment of esophageal cancer over surgery alone by showing improved OS, PFS, and an outstanding rate of pathologic response in patients treated with CRT compared to those treated with RT alone [2]. This significant benefit was realized despite using a de-escalated dose of 41.4 Gy, which represents an almost 20% decrease from the 50.4 Gy which was standardly used in the United States. These findings raise significant questions regarding the optimization of the therapeutic ratio in the treatment of esophageal cancer, as physicians attempt to achieve the highest rates of the cure while minimizing patient toxicity and the use of healthcare resources. Due to the complexities and cost of designing and executing a non-inferiority randomized trial aimed at comparing these two dose regimens, decision-making will likely continue to be based on available retrospective data and physician preference. 

The data from our institution did not show any significant difference in pCR rates between the two cohorts; additionally, no differences in OS or PFS were detected. This was despite the fact that a greater proportion of patients treated with 41.4 Gy had AC, a subset of patients who have classically suffered from poorer outcomes on studies like the CROSS trial [10]. This may be partially due to the relatively high pCR rate (47.1%) in patients with AC in the 41.4 Gy cohort of our study, which was likely a consequence of the small overall number of patients in each subgroup. Otherwise, our data appears to be in congruence with the large population-based study by Li et al. [16]. This National Cancer Database (NCDB) study of 7,996 patients suggested equivalent three-year OS, 90-day mortality, and risk of positive margins in patients treated with 41.4 - 45 Gy compared to 50 - 54 Gy. However, there was a slightly higher pCR rate in the 50 - 54 Gy group compared to the 41.4 - 45 Gy group (20.3% vs 16.3%). These results are similar to other NCDB data that showed no survival differences across dose levels [17-18]. 

There are two competing arguments for determining the appropriate dose for treating patients with neoadjuvant intent. One side would argue that using lower doses of radiation may decrease toxicity and lower the rate of complications postoperatively. Although not validated by statistical analysis, our study showed a numerical increase in grade 3 toxicity in patients treated with 50.4 Gy compared to those treated with 41.4 Gy. Additionally, although also not statistically different, there was a large decrease in the frequency of postoperative complications seen in the 41.4 Gy group, perhaps implying that a significant difference may be seen in larger cohorts. These points may suggest that, given there appears to be no improvement in clinical outcomes between the two doses in our data, 50.4 Gy may increase toxicity and complication rates without providing a significant benefit to patients. It is important to note, however, despite the slightly longer treatment time and the trend towards increased postoperative complications, treatment with 50.4 Gy did not appear to increase the overall treatment time compared to treatment with 41.4 Gy.

On the other hand, it can be argued that treating patients with doses less than 50.4 Gy, largely considered the “definitive” radiation dose for esophageal cancer, could lead to poor outcomes in patients who do not ultimately undergo oncologic resection. Although outside of the scope of our study, retrospective data shows improved local control rates with higher doses of radiation in patients who were treated with definitive CRT [19-20]. Additional retrospective and population-based studies show that there may also even be a survival benefit to higher doses for patients treated with definitive CRT or for those being treated with neoadjuvant intent that did not subsequently undergo oncologic resection [16, 21]. However, given that 90% of patients on the CRT arm of the CROSS trial underwent resection after completing RT, it remains unclear how much of an effect this would have on a larger scale [2].

Our study has a number of limitations. Most importantly, this was a retrospective analysis, making it impossible to account for a number of biases that could have affected our results. The number of patients available for analysis was very small. The doses prescribed to each patient were not given in a randomized fashion and were likely influenced by the time period in which the patient was treated (before or after the CROSS trial data was available) and the likelihood that they would be able to tolerate a surgical resection following neoadjuvant CRT. Additionally, while the two cohorts were relatively well-balanced, there was a significantly higher rate of IMRT and prevalence of AC in the group treated with 41.4 Gy. The increased use of IMRT could have potentially improved outcomes with a more conformal treatment plan and may have contributed to the lower rate of late toxicity observed, although that value was not significant. This idea is supported by previous studies showing improved outcomes and dosimetry in patients treated with IMRT compared to two-dimensional (2D) and 3D techniques [22-23]. The increased prevalence of AC in the 41.4 Gy cohort may have decreased the rate of pCR in that group, based on the historically lower rates of pCR in patients with AC compared to SCC [10]. Although our analysis did not identify statistically significant differences in pCR between patients with SCC and AC, there were large numerical differences in the pCR rates we observed that may have been significant with a larger number of patients. This concept is validated by the CROSS trial, in which the pCR rates for AC (23%) were much lower than the rate for SCC (49%) [10]. It should also be noted that the total pCR rate in our study (44.2%) was much higher than what was observed on the CROSS trial (29%). Finally, the use of carboplatin and paclitaxel was significantly increased in the 41.4 Gy arm, which could have altered the effectiveness of the overall treatment and affected the pCR rates in our study. This was likely related to the higher likelihood of being treated per the CROSS trial (41.4 Gy with carboplatin and paclitaxel) after its publication in 2015. However, it should be noted that, although this has not been studied in a randomized fashion, retrospective data suggest that there is no significant difference between carboplatin plus paclitaxel and other regimens in the neoadjuvant or definitive setting [24-25]. While a randomized clinical trial may never be conducted comparing 50.4 Gy and 41.4 Gy in the neoadjuvant setting, larger cohorts or patients with prospectively collected data are needed to confirm our results and control for many of the biases observed in our study.

## Conclusions

Our study aimed to use retrospective institutional data to determine whether there is an advantage to treating locally advanced but resectable esophageal cancer with a “definitive” dose of 50.4 Gy when compared to 41.4 Gy. Our results demonstrated no improvement in pCR rates, OS, or PFS in patients treated with 50.4 Gy compared to those treated with 41.4 Gy, despite higher rates of patients with AC in the 41.4 Gy cohort. Additionally, there was a numerical increase in late grade 3 toxicity and postoperative complications seen in patients treated with 50.4 Gy compared to those treated with 41.4 Gy. This data suggests that, in selected patients for whom surgical resection is highly likely, 41.4 Gy may provide equivalent oncologic outcomes with the possibility of less late adverse effects. However, the dose of radiation delivered should be made on a case-by-case basis to ensure that all patient and clinical factors are accounted for. 
